# Improved survival at the cost of more chronic lung disease? Current management and outcomes in extremely preterm infants born in New South Wales and the Australian Capital Territory: 2010–2020

**DOI:** 10.1007/s12519-023-00761-3

**Published:** 2023-10-30

**Authors:** Nele Legge, Himanshu Popat, Dominic Fitzgerald

**Affiliations:** 1 Liverpool Hospital, Corner Elizabeth and Goulburn Streets, Liverpool, NSW Australia; 2 University of Sydney, Camperdown, Australia; 3 Children’s Hospital Westmead, Westmead, Australia

**Keywords:** Chronic lung disease, Extreme prematurity, Health outcomes, Survival

## Abstract

**Background:**

Since 2010, most tertiary care hospitals in Australia have changed how they care for extremely premature infants. However, in-hospital and longer-term outcome data have suggested unchanged or even worse health outcomes in later epochs, especially respiratory outcomes. This study examined the trend in outcomes since these changes were introduced, particularly the prevalence of chronic neonatal lung disease (CLD).

**Methods:**

This is a retrospective cross-sectional analysis of data from the Neonatal Intensive Care Units’ (NICUS) database of all perinatal intensive care units in New South Wales and the Australian Capital Territory, including infants born at ≥ 24 and ≤ 28 weeks of gestational age in tertiary perinatal units between January 1, 2010, and December 31, 2020. Temporal trends and changes in primary outcome were examined by linear and adjusted multivariable logistic regression models.

**Results:**

This study included 3258 infants. We saw significant changes in antenatal magnesium sulfate (75% increase), delayed cord clamping (66% increase), delivery room intubations (30% decrease), any time (20% decrease), duration on mechanical ventilation (100-hour decrease), and hours on noninvasive ventilation (200-hour increase). Mortality decreased from 17% to 6%. The incidence of CLD increased significantly even when adjusted for confounders (15% increase). Any time and mean hours spent on mechanical ventilation significantly increased the odds of CLD. This study could not find a significant association of any of the protective antenatal treatments on CLD.

**Conclusions:**

The last decade saw a significant improvement in survival and survival to discharge without major morbidity. There was increased use of magnesium sulfate, delayed cord clamping, and less invasive respiratory management of extremely preterm infants. The avoidance of mechanical ventilation may impact the incidence of CLD.

## Introduction

Over 15 million infants worldwide were born prematurely in 2020, 25,000 of whom were born in Australia [1, 2]. Preterm birth complications are the leading cause of death in children younger than five years. Over the past 20 years, there has been a shift in the management of extremely preterm infants worldwide. The focus started to change from merely increasing survival to improving quality of life and preventing adverse long-term outcomes in survivors. One comorbidity that has been described as having a long-term impact on the child’s quality of life is chronic neonatal lung disease (CLD) [3]. Many changes in treatment and management are therefore aimed at reducing CLD.

The use of antenatal corticosteroids for threatened preterm labor was introduced after Liggins’ study in 1972, leading to significantly better survival and respiratory outcomes [3]. Studies in the late 1990s showed that maternal antenatal administration of magnesium sulfate (MgSO_4_) in very preterm babies can decrease the incidence of cerebral palsy [4, 5], leading to the introduction of routine antenatal administration of MgSO_4_ where preterm delivery is imminent in developed countries, including Australia.

Many trials have shown that the avoidance of intubation at birth and the preference for continuous positive airway pressure (CPAP) lead to better outcomes in preterm infants, namely a decrease in mortality and CLD, most notably the 2011 “Continuous Positive Airway Pressure or Intubation at Birth” (COIN) trial [6]. Following this, protocols aiming to avoid intubation in the delivery room in favor of noninvasive ventilation strategies have emerged [7, 8]. To avoid intubation but still be able to provide life-saving surfactant, an increasing number of infants receive surfactant via less invasive administration techniques. Multiple studies have shown improved rates of CLD and survival utilizing these techniques [7, 8].

The Australian Placental Transfusion Study (APTS), completed in 2017, showed that delayed clamping of the cord for at least 60 seconds to increase the circulating blood volume reduced the risk of death or major morbidity at two years [9]. This practice is slowly being adopted across Australian perinatal centers.

Significant clinical practice changes over the last decade have included antenatal maternal MgSO_4_ administration, delayed cord clamping and increased prioritization of early noninvasive ventilation. No recent audit has been undertaken on the translation of these studies into everyday practice and their impact on early outcomes in Australia.

This paper will describe the changes over time and coinciding changes in outcomes, primarily in the incidence of CLD, in a decade when we cared for ever smaller and younger infants trying to learn how doing “less is more.”

## Methods

This is a retrospective cross-sectional analysis of prospectively collected data from all perinatal, tertiary neonatal intensive care units (NICUs) in New South Wales and the Australian Capital Territory, Australia. Ethical approval was obtained from the South Western Sydney Local Health District Human Research Ethics Committee (2021/ETH01027).

The study population included all infants born at ≥ 24 and ≤ 28 weeks of gestational age (GA) admitted to tertiary neonatal units between January 1, 2010, and December 31, 2020. Infants with major congenital malformations, those born outside a tertiary NICU, and those weighing < 500 g at birth were excluded from this study. Infants who died prior to reaching 36 weeks postmenstrual age (PMA) were described in overall survival numbers but excluded from further analysis since CLD measured as oxygen requirement at 36 weeks PMA was an endpoint in the analysis.

Clinical data for this study were sourced from the prospectively collected Neonatal Intensive Care Units’ (NICUS) Data Collection, an ongoing regional audit of live-born neonates admitted to tertiary NICUs in New South Wales and the Australian Capital Territory. These data were audited by dedicated NICUS Audit Officers.

Data points collected and analyzed included relevant demographic data on the patient (GA, birthweight, sex, parity) and mother (maternal age, diabetes status), as well as perinatal risk factors [intrauterine growth restriction (IUGR), chorioamnionitis, prolonged rupture of membranes, mode of delivery] and perinatal therapies relevant to the patient [antenatal antibiotics, corticosteroids, magnesium sulfate, delayed cord clamping (DCC)]. Postnatal data included important early [intraventricular hemorrhage (IVH), periventricular leukomalacia (PVL), retinopathy of prematurity (ROP), necrotizing enterocolitis (NEC), surgical ligation of persistent ductus arteriosus (PDA)] and late in-hospital outcomes (chronic lung disease, defined as oxygen or positive pressure requirement at 36 weeks PMA, home oxygen/respiratory support, survival to 36 weeks PMA, and length of stay) and common treatments in the hospital (presence and duration of mechanical ventilation and noninvasive respiratory support at birth and beyond, surfactant, postnatal steroids). Analyzing survival to discharge without major morbidity included patients discharged alive without home oxygen therapy, IVH, PVL, surgical NEC, surgical ROP, or requiring ligation of the PDA.

Means were compared using ANOVA. Differences in categorical data were assessed using chi-squared tests. All *P* values were two-sided. Statistical significance was assumed at *P* < 0.05. Logistic regression was used for binary outcomes. Temporal changes were examined using line graphs and linear regression for continuous variables. To evaluate which variables had a significant effect on CLD, defined as oxygen requirement at 36 weeks PMA, we performed binary logistic regression of variables of interest. Significant variables were then included in the multivariable logistic regression model to control for confounding bias adjustments and expressed as adjusted odds ratios (aOR) with 95% confidence intervals (CI). SPSS Version 28 (IBM, New York, USA, 2021) was used for all calculations.

The authors had full access to all the data (including statistical reports and tables) in the study. Individual participant data that underlie the results reported in this article, after de-identification (text, tables, figures, and appendices), can be made available on request.

## Results

Of 3703 babies who met the inclusion criteria, 3296 (89%) infants survived until 36 weeks PMA. Their mean gestational age at birth was 26 weeks, and the mean birthweight was 947 g. Over time, there was a statistically significant decrease in gestational age (GA) from a minimum of 26.8 weeks in 2011 to a maximum of 26.4 weeks in 2018 (*P* ≤ 0.001); however, there was no statistically significant change in birthweight. There was a significant increase in the proportion of infants born between 24 and 26 weeks of GA who survived to 36 weeks postmenstrual age, from 17% in 2010 to 26% in 2020 (*P* ≤ 0.001) (Table 1).

**Table 1:** Demographics of the study population

Variables	2010 (260)	2011 (317)	2012 (294)	2013 (307)	2014 (315)	2015 (299)	2016 (330)	2017 (314)	2018 (283)	2019 (303)	2020 (274)	*P*
Birthweight, g (mean ± SD)	968 ± 212	986 ± 247	968 ± 228	962 ± 220	975 ± 220	985 ± 227	954 ± 225	947 ± 217	949 ± 218	944 ± 216	973 ± 232	N.S
Gestational age, (mean ± SD)	26.7 ± 1.2	26.8 ± 1.3	26.7 ± 1.2	26.7 ± 1.3	26.8 ± 1.3	26.8 ± 1.3	26.5 ± 1.3	26.5 ± 1.3	26.4 ± 1.3	26.5 ± 1.3	26.5 ± 1.3	< 0.001
< 26/40, *n* (%)	45 (17%)	59 (19%)	50 (17%)	56 (18%)	60 (19%)	52 (17%)	76 (23%)	82 (26%)	74 (26%)	77 (25%)	70 (26%)	< 0.001
Gender, male (%)	141 (55%)	162 (52%)	163 (56%)	154 (51%)	170 (54%)	150 (52%)	188 (58%)	159 (51%)	162 (58%)	160 (53%)	144 (53%)	N.S
Singleton, *n* (%)	183 (71%)	231 (74%)	221 (76%)	214 (71%)	232 (74%)	203 (70%)	238 (73%)	209 (67%)	213 (76%)	221 (74%)	194 (71%)	N.S
Cesarean section, *n* (%)	152 (59%)	203 (65%)	176 (60%)	183 (61%)	191 (61%)	192 (67%)	207 (63%)	215 (69%)	184 (65%)	209 (69%)	195 (71%)	< 0.001
Apgar 1’, median (IQR)	6 (3)	6 (3)	6 (3)	6 (3)	6 (3)	6 (4)	5 (4)	6 (3)	6 (3)	6 (3)	6 (4)	N.S
Apgar 5’, median (IQR)	8 (2)	8 (2)	8 (2)	8 (2)	8 (2)	8 (2)	8 (3)	8 (2)	8 (2)	8 (2)	8 (3)	N.S
Intrauterine growth restriction, *n* (%)	18 (6.9%)	25 (5.7%)	28 (9.5%)	26 (8.5%)	18 (5.7%)	20 (6.7%)	22 (6.7%)	21 (6.7%)	17 (6%)	26 (8.6%)	11 (4%)	N.S
Maternal antibiotics, *n* (%)	149 (57%)	182 (58%)	179 (62%)	197 (65%)	206 (66%)	186 (65%)	202 (62%)	194 (62%)	194 (69%)	219 (73%)	198 (72%)	< 0.001
Maternal diabetes, *n* (%)	19 (7.4%)	31 (10%)	30 (10.3%)	35 (11.6%)	40 (12.7%)	45 (15.6%)	51 (15.6%)	59 (18.8%)	41 (14.5%)	56 (18.6%)	39 (14.2%)	< 0.001
Maternal age (mean ± SD)	30.3 ± 6.5	30 ± 6	31 ± 6.6	30.4 ± 6.5	31.2 ± 5.9	30.3 ± 5.9	30.7 ± 6	31.5 ± 6	31.2 ± 5.9	31.8 ± 5.7	31.4 ± 5.2	< 0.001
MgSO_4_, *n* (%)	22 (8.5%)	50 (16%)	106 (36%)	169 (56%)	198 (63%)	208 (72%)	236 (72%)	240 (76%)	214 (76%)	245 (82%)	231 (84%)	< 0.001
Chorioamnionitis, *n* (%)	75 (29%)	82 (26%)	95 (32%)	115 (38%)	114 (36%)	129 (45%)	141 (43%)	115 (37%)	131 (47%)	118 (39%)	121 (44%)	< 0.001
Prolonged rupture of membranes > 18 h, *n* (%)	77 (30%)	101 (32%)	98 (34%)	81 (27%)	90 (29%)	102 (35%)	94 (29%)	86 (27%)	92 (33%)	110 (37%)	101 (37%)	0.041
Delayed cord clamping, *n* (%)	0	0	0	10 (3%)	50 (16%)	43 (15%)	52 (16%)	72 (23%)	170 (60%)	199 (66%)	181 (66%)	< 0.001
Delivery room intubation, *n* (%)	173 (67%)	196 (62%)	168 (57%)	190 (62%)	190 (60%)	151 (51%)	161 (49%)	123 (39%)	115 (41%)	120 (40%)	104 (38%)	< 0.001
Surfactant, *n* (%)	237 (91%)	272 (86%)	255 (87%)	261 (85%)	249 (79%)	234 (78%)	268 (81%)	255 (81%)	231 (81%)	253 (83%)	224 (81%)	< 0.001
Antenatal steroids, *n* (%)												N.S
None	11 (4%)	16 (5%)	13 (4%)	19 (6%)	9 (3%)	6 (2%)	13 (4%)	8 (3%)	6 (2%)	6 (2%)	12 (4%)	
< 24 h	85 (33%)	75 (24%)	78 (27%)	74 (25%)	86 (27%)	81 (28%)	82 (26%)	85 (27%)	71 (25%)	65 (22%)	70 (26%)	
Complete	107 (41%)	163 (52%)	151 (52%)	167 (55%)	153 (49%)	142 (48%)	171 (52%)	172 (55%)	164 (58%)	186 (62%)	161 (59%)	
> 7d	55 (21%)	58 (19%)	49 (17%)	41 (14%)	66 (21%)	59 (20%)	60 (18%)	48 (15%)	40 (14%)	43 (14%)	31 (11%)	

*N.S.* nonsignificant (*P* > 0.05), *SD *standard deviation, < *26/40* infants born at less than 26 completed weeks of gestational age, *IQR* interquartile range, *MgSO*_*4*_ magnesium sulfate

There was no difference in the incidence of male sex, intrauterine growth restriction (IUGR), or singleton births. Maternal age at delivery increased over time from a minimum mean of 29.9 years in 2011 to a maximum of 31.8 years in 2019 (*P* ≤ 0.001). The rate of women diagnosed with any type of diabetes mellitus (type 1, type 2, and gestational diabetes) significantly increased over time from a minimum of 7.4% diagnosed in 2010 to a maximum of 18.8% in 2019 (*P* ≤ 0.001). The rate of premature prolonged rupture of membranes and the incidence of chorioamnionitis increased significantly over time. The use of antenatal antibiotics increased significantly from a minimum of 57% in 2010 to a maximum of 73% in 2019 (*P* ≤ 0.001). There was a significant increase in babies delivered by cesarean section from 59% in 2010 to 71% in 2020 (*P* ≤ 0.001) (Table 1).

Coverage with antenatal corticosteroids (two doses prior to delivery) increased nonsignificantly from a minimum of 62% in 2010 to a maximum of 76% in 2019 (*P* = 0.75). Antenatal treatment with MgSO_4_ for neuroprotection increased significantly from 8.5% in 2010 to 84% in 2020 (*P* ≤ 0.001). There was a significant increase in delayed cord clamping from 0% in 2010 to 66% in 2020 (*P* ≤ 0.001). The rate of babies intubated at birth decreased significantly from 67% in 2010 to 38% in 2020 (*P* ≤ 0.001) (Table 1).

**Fig. 1:**
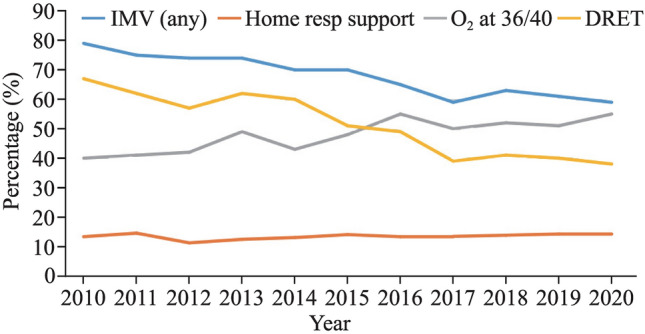
Changes in respiratory management of the study population. *IMV *invasive mechanical ventilation, *home resp support*—respiratory support after discharge, *O*_*2 *_oxygen, *36/40* 36 weeks postmenstrual age, *DRET*—delivery room endotracheal tube

There was a consistently high use of surfactant in the NICU, with over 80% of patients receiving surfactant. However, the number of babies receiving surfactant decreased over time from 91% to 81% (*P* ≤ 0.001). The duration of mechanical ventilation decreased from a maximum of 189 hours in 2010 to a minimum of 88 hours in 2019 (*P* ≤ 0.001). Hours on noninvasive ventilation increased significantly, with a mean of 967 hours in 2010 to 1362 hours in 2020 (*P* ≤ 0.001). Similarly, hours on high-flow nasal prong (HFNP) increased from < 200 hours in 2010 to > 300 hours in 2020 (*P* ≤ 0.001). The proportion of babies that received any mechanical ventilation decreased significantly from 79% to 59% (*P* ≤ 0.001), while treatment with postnatal steroids for CLD increased significantly from 16% in 2010 to a maximum of 25% in 2020 (*P* ≤ 0.001). The proportion of babies discharged home on oxygen or CPAP remained stable at approximately 13% (Table 2). Figure 1 depicts changes over time in respiratory management of the study population.

**Table 2:** Change in respiratory treatments over the study period

Variables	2010 (260)	2011 (317)	2012 (294)	2013 (307)	2014 (315)	2015 (299)	2016 (330)	2017 (314)	2018 (283)	2019 (303)	2020 (274)	*P*
Mechanical ventilation, *n* (%)	204 (79%)	237 (75%)	208 (71%)	226 (74%)	221 (70%)	210 (70%)	216 (65%)	185 (59%)	178 (63%)	185 (61%)	163 (59%)	< 0.001
Hours on mechanical ventilation (mean ± SD)	189 ± 331	160 ± 278	114 ± 220	158 ± 314	101 ± 217	132 ± 282	113 ± 235	86.7 ± 170	96 ± 183	88 ± 195	113 ± 276	< 0. 001
Hours on noninvasive ventilation (mean ± SD)	967 ± 630	960 ± 625	1060 ± 675	1097 ± 607	1089 ± 625	1214 ± 675	1300 ± 671	1291 ± 560	1290 ± 559	1362 ± 581	1362 ± 577	< 0.001
Hours on CPAP (mean ± SD)	761 ± 522	738 ± 498	772 ± 485	695 ± 435	689 ± 486	751 ± 530	863 ± 493	843 ± 438	843 ± 438	917 ± 426	878 ± 437	< 0.001
Hours on high flow (mean ± SD)	175 ± 254	178 ± 275	243 ± 303	354 ± 380	350 ± 300	390 ± 378	319 ± 392	304 ± 300	304 ± 300	317 ± 337	342 ± 316	< 0.001
Postnatal steroids, *n* (%)	42 (16%)	52 (16%)	44 (15%)	66 (22%)	35 (11%)	59 (20%)	55 (17%)	52 (17%)	68 (24%)	72 (24%)	68 (25%)	< 0.001
Oxygen at 36/40 postmenstrual age, *n* (%)	105 (40%)	131 (41%)	123 (42%)	149 (49%)	135 (43%)	143 (48%)	181 (55%)	158 (50%)	147 (52%)	156 (51%)	151 (55%)	< 0.001
Home oxygen, *n* (%)	39 (15%)	40 (13%)	27 (9%)	36 (12%)	25 (8%)	36 (12%)	38 (12%)	38 (12%)	34 (12%)	35 (12%)	37 (14%)	N.S
Home CPAP, *n* (%)	1 (0.4%) %)	3 (0.9%)	3 (1%) %)	1 (0.3%)	8 (2.5%)	3 (1%) %)	3 (0.9%)	2 (0.6%)	3 (1.1%)	4 (1.3%)	1 (0.4%)	N.S
Any home respiratory support, *n* (%)	40 (15%)	43 (14%)	30 (10%)	37 (12%)	33 (10%)	39 (13%)	41 (12%)	40 (13%)	37 (13%)	39 (13%)	38 (14%)	N.S

*N.S.* nonsignificant (*P* > 0.05), *SD* standard deviation, *CPAP* continuous positive airway pressure

The incidence of neonatal CLD increased significantly over time from 40% in 2010 to 55% in 2020 (*P* ≤ 0.001). The number of infants who suffered a major intraventricular hemorrhage (IVH) (grade III or grade IV) or periventricular leukomalacia (PVL) and survived to 36 weeks PMA was small—only 106 for IVH and 78 for PVL. Among the survivors, there were year-on-year fluctuations with no statistically significant change over time.

There was no significant difference in the average length of hospital stay (range: 82–89 days). The rate of surgical treatment for retinopathy of prematurity (ROP) or necrotizing enterocolitis (NEC) was stable at 8% and 2.5%, respectively. The rate of ligation surgery for persistent ductus arteriosus (PDA) significantly decreased from a maximum of 9.2% in 2010 to a minimum of 1.3% in 2019 (*P* ≤ 0.001) (Table 3).

**Table 3:** Major neonatal morbidities over the study period

Variables	2010 (260)	2011 (317)	2012 (294)	2013 (307)	2014 (315)	2015 (299)	2016 (330)	2017 (314)	2018 (283)	2019 (303)	2020 (274)	*P*
Major IVH, *n* (%)	17 (6.5%)	4 (1.3%)	15 (5.1%)	8 (2.6%)	10 (3.2%)	9 (3%)	13 (3.9%)	11 (3.5%)	7 (2.5%)	9 (3%)	9 (3.3%)	N.S
PVL, *n* (%)	15 (5.8%)	4 (1.3%)	10 (3.4%)	4 (1.3%)	6 (1.9%)	2 (0.7%)	10 (3%)	5 (1.6%)	11 (3.9%)	7 (2.3%)	8 (2.9%)	N.S
Survival to 36 weeks PMA, *n* (% of whole cohort)	260/315 (83%)	317/365 (87%)	294/330 (89%)	307/345 (89%)	315/351 (90%)	299/333 (90%)	330/363 (91%)	314/352 (89%)	283/320 (88%)	303/337 (90%)	274/292 (94%)	< 0.001
Length of stay, d, (mean ± SD)	86 ± 33	83 ± 37	85 ± 41	86 ± 37	82 ± 40	85 ± 32	89 ± 36	89 ± 31	89 ± 30	89 ± 31	89 ± 30	N.S
Severe ROP requiring treatment, *n* (%)	21 (8%)	30 (9.5%)	23 (7.8%)	22 (7.2%)	24 (7.6%)	16 (5.4%)	26 (7.9%)	26 8(.2%)	26 (9.2%)	26 (8.5%)	20 (7.3%)	N.S
Surgical NEC, *n* (%)	10 (3.8%)	5 (1.6%)	7 (2.4%)	7 (2.3%)	5 (1.6%)	9 (3%)	7 (2.1%)	5 (1.6%)	6 (2.1%)	10 (3.3%)	4 (1.5%)	N.S
PDA ligations, *n* (%)	24 (9.2%)	18 (5.7%)	18 (6.1%)	17 (5.5%)	16 (5%)	11 (3.7%)	10 (3%)	6 (1.9%)	10 (3.5%)	4 (1.3%)	4 (1.5%)	< 0.001
Survival without major morbidity, *n*	163 (63%)	228 (72%)	211 (72%)	225 (73%)	247 (78%)	222 (74%)	241 (73%)	238 (76%)	202 (71%)	232 (77%)	206 (75%)	N.S

*N.S.* nonsignificant (*P* > 0.05), *SD* standard deviation, *IVH* intraventricular hemorrhage, *PVL* periventricular leukomalacia, *ROP* retinopathy of prematurity, *NEC* necrotizing enterocolitis, *PDA* persistent ductus arteriosus, *PMA* postmenstrual age

**Fig. 2:**
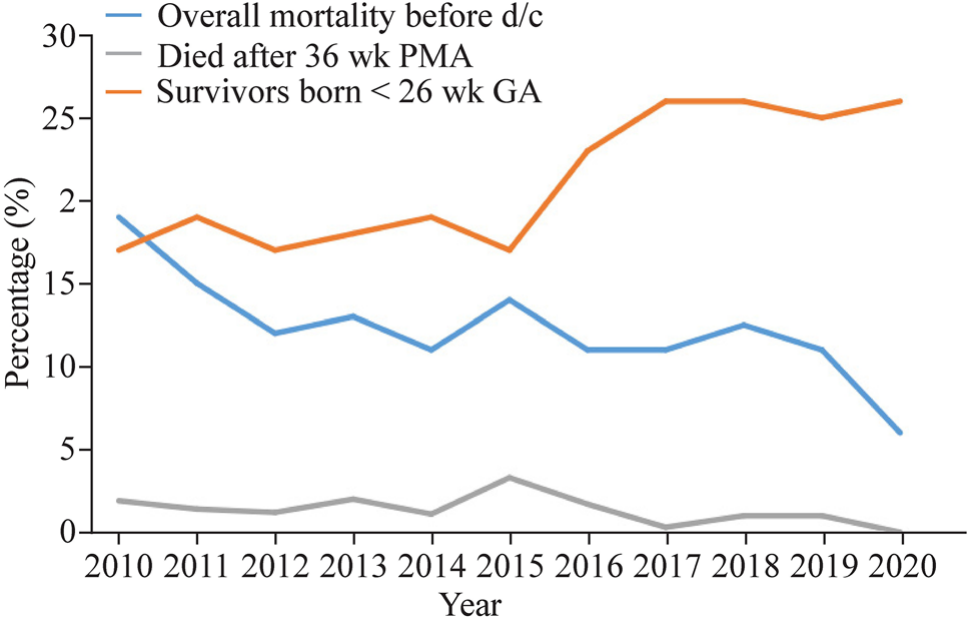
Changes in survival to 36 weeks PMA for infants born at < 26/40 and whole cohort mortality. *26/40* gestational age under 26 weeks, *GA* gestational age, *PMA* postmenstrual age, d/c discharge

Survival to 36 weeks PMA increased significantly. In 2010, 83% of babies born ≤ 28 weeks of GA who were admitted to the NICU survived to 36 weeks PMA, whereas in 2020, that number increased to 94%. Fifty babies died after 36 weeks PMA and were included in our analysis but did not leave the hospital. Almost half of these late deaths were due to CLD (23 died of CLD, six of bowel complications, five of sepsis/meningitis, four of sudden infant death syndrome, three of cerebral ischemia, two of pneumonia, and seven of a variety of other conditions)). Figure 2 shows a graphic description of overall survival to 36 weeks PMA and the proportion of infants born at < 26 weeks of GA.

We analyzed survival to discharge without major morbidity and found stable rates, with 2010 being an outlier at 63%, and the years following that ranged from about 72% to 78%, with no significant difference between 2011 and 2020.

Logistic regression found a significantly higher odds ratio for the incidence of CLD with decreasing GA and birthweight, presence of IUGR, male gender, low-one minute Apgar, and year of birth. We continued using these parameters as potential confounders and found a significantly increased adjusted odds ratio for CLD in patients who were intubated in the delivery room, spent any time on mechanical ventilation, remained ventilated for a long duration and spent any time on HFNP, and those who received surfactant. We found no difference in adjusted odds ratio (aOR) for CLD with antenatal steroids, MgSO_4_, delayed cord clamping, or any of the maternal risk factors (Table 4).

**Table 4:** Multivariable logistic regression of factors associated with chronic lung disease

Variables	aOR (95% CI)	*P* value
Gestational age (wk)	0.68 (0.625–0.749)	< 0.001
Birthweight (g)	0.99 (0.997–0.998)	< 0.001
Year of birth	1.04 (1.036–1.091)	< 0.001
Gender (male)	1.65 (1.403–1.941)	< 0.001
IUGR	3.14 (2.048–4.812)	< 0.001
Delivery room intubation	1.21 (1.025–1.422)	0.023
Apgar 1	0.89 (0.855–0.92)	< 0.001
Any mechanical ventilation	1.66 (1.38–1.988)	< 0.001
Hours of mechanical ventilation	1.01 (1.004–1.006)	< 0.001
Any time on nasal high flow	2.05 (1.715–2.444)	< 0.001
Surfactant	1.79 (1.406–2.27)	< 0.001

*IUGR* intrauterine growth restriction, *aOR* adjusted odds ratio, *CI* confidence interval

## Discussion

Our study showed an increase in all maternal interventions over the last decade. There was an increase in antenatal risk factors, including maternal age, diabetes, chorioamnionitis, premature prolonged rupture of membranes, antibiotic treatment, and cesarean sections. Protective treatment in the form of antenatal steroids and MgSO_4_ increased, as did the practice of delayed cord clamping. While there was an increasing number of smaller and younger infants resuscitated and admitted to the NICU, this study showed that significantly fewer infants were intubated at resuscitation. The use of mechanical ventilation decreased; noninvasive ventilation increased, and mortality decreased from 17% to 6%.

Reassuringly, most in-hospital outcomes remained stable or improved. Fewer babies underwent surgery for PDA, and stable numbers of surgeries were required for NEC and ROP. The length of hospital stay also remained stable. The incidence of major IVH and PVL in survivors remained the same, as did infants who required respiratory support at home. Taken together, these outcomes showed stable rates of survival without major morbidity. All these outcomes remained stable despite a significant increase in the number of babies born before 26 weeks of gestational age and a steady rise in antenatal risk factors.

Evaluating factors associated with CLD, we found that any parameter related to mechanical ventilation increased the patients’ odds of developing CLD. Of note, there was a significant association between any time spent on HFNP and the likelihood of developing CLD. Administration of surfactant was unexpectedly associated with increased odds of developing CLD. Also demonstrated was the lack of association between any of the antenatal risk factors and treatments, such as chorioamnionitis, antenatal steroids, and developing CLD.

The changes in maternal health were striking, with an increase in almost all antenatal risk factors for adverse neonatal outcomes. Some of these results can be explained by looking at the shift in maternal demographics such as age and weight [10]. Other considerations may relate to increasing obstetric surveillance and more obstetric interventions to prevent preterm birth, such as cervical cerclages and better and more rigorous ways to diagnose conditions such as gestational diabetes and chorioamnionitis [11]. Furthermore, over time, more infants in the lower gestational age bracket were resuscitated and survived, especially infants who were not offered resuscitation and did not survive and infants whose mothers often presented with more risk factors at birth.

Finding an increase in CLD in our population was disappointing. However, measuring CLD as a dichotomous outcome of oxygen requirement at a certain age has been debated for some time now [12, 13]. Other studies have reported a stagnant or even increasing incidence of CLD over time [14, 15]. Interestingly, in our study, the rate of infants discharged on oxygen or CPAP remained stable despite a population where more lower gestational age infants have survived, infants who are more susceptible to adverse respiratory outcomes.

The association of surfactant administration and CLD was unexpected. However, multiple studies failed to show a decrease in CLD with routine surfactant administration [16, 17]. Surfactant could have played a role in keeping sicker and smaller patients alive who then developed CLD due to their burden of risk factors (i.e., lower gestational age, etc.).

The correlation between HFNP and CLD has been described previously. Taha et al. in 2016 reported increased odds for CLD, death, prolonged ventilation days, and length of stay in a retrospective audit of over 2000 extremely low birthweight infants receiving HFNP [18]. However, one could also argue that high flow was used as part of noninvasive ventilation in infants that in the past would have been mechanically ventilated.

Other neonatal networks have reported on their longitudinal outcomes in various epochs. Stoll et al. reported on the outcomes in Neonatal Research Network Centers in the USA and, similar to our analysis, found improved survival, an increase in CLD and obstetric interventions, and a decrease in mechanical ventilation [14]. More recently, Boel et al. presented outcomes of extremely preterm infants in the UK and found a decrease in the utilization of mechanical ventilation and an increase in the use of HFNP. They reported stable rates of IVH, CLD, ROP, and NEC [19]. A major limitation of their study and especially their results of CLD is over 60% missing data for CLD diagnosis. Additionally, in 2020, the Neonatal Network from South America (NEOCOSUR) reported more obstetric intervention, increased antenatal corticosteroids, and an avoidance of mechanical ventilation in favor of noninvasive ventilation techniques. Their rates of IVH and PVL remained stable, similar to our results. They observed a very slight decrease in the incidence of CLD in their population [20]. Comparisons to this study’s results of CLD are difficult, as their study population included all infants up to 35 weeks of gestational age.

There are several strengths to our study. Our population was well defined. It is a geographical cohort and likely to accurately reflect the Australian extremely preterm population. All data used were prospectively added to a purpose-built database and were audited by trained audit officers prior to analysis, minimizing information bias. Our results are detailed, longitudinal, without any missing data, and adjusted for confounders, and we report a confidence interval for all outcomes and show robust *P* values.

Our study has several limitations. Epidemiological studies cannot determine causation, and our analysis can only describe associations of changes in practice and outcomes. Furthermore, due to the design of the study, our population does not include all extreme preterm patients being cared for in the last decade. The number of babies resuscitated at < 24 weeks of gestation and < 500 g birthweight is steadily increasing but was very low in the early 2010s [21]. Furthermore, the treatment and management of these babies are still widely variable in the included eight NICUs. Including these babies would have led to significant outliers and affected the validity of our results. Our study also cannot control changes in diagnostic criteria or management policies; we can only describe the change in practice that is measured. One of these changes could have influenced the incidence of some outcome measures, including our primary outcome—chronic lung disease. Changes in oxygen targets were slowly implemented during the study period as a result of randomized controlled trials such as BOOSTII, leading to higher targets and consequently potentially more oxygen use [22]. One further limitation is the inclusion of 2020. The global pandemic has had an impact on the incidence of preterm birth worldwide [23]. Our study cannot account for the impact the global pandemic had on the management and outcomes of our patients.

In summary, we identified that a clinical practice shift toward less invasive ventilation and avoidance of mechanical ventilation was temporally associated with an unchanged incidence of home respiratory support despite increasing antenatal risk factors, decreasing gestational age, and increasing survival. When providing less invasive ventilation, the use of HFNP should be considered with caution, as our results showed a potential to increase the odds of developing CLD in this population. No other change in management had a significant impact on our primary outcome. This study did not examine the impact of these measures on mortality, an outcome that significantly decreased over the last decade. Reassuringly, the incidence of major secondary outcomes such as ROP, NEC, PDA surgery, and length of stay either remained stable or decreased despite a steady decrease in gestational age. The results of this study add to the ever-growing body of evidence indicating that a shift away from mechanical ventilation might improve respiratory morbidity. Since CLD was by far the most important contributor to mortality after 36 weeks PMA, clinicians should consider these results in their daily practice.

## Data Availability

The authors had full access to all the data (including statistical reports and tables) in the study. Individual participant data that underlie the results reported in this article after de-identification (text, tables, figures, and appendices) can be made available on request.
